# Early Psychological Impact of the COVID-19 Pandemic in Brazil: A National Survey

**DOI:** 10.3390/jcm9092976

**Published:** 2020-09-15

**Authors:** Juliana Alvares Duarte Bonini Campos, Bianca Gonzalez Martins, Lucas Arrais Campos, João Marôco, Rayya Ahmed Saadiq, Rodrigo Ruano

**Affiliations:** 1 School of Pharmaceutical Sciences, São Paulo State University (UNESP), Campus Araraquara, São Paulo 14800-903, Brazil; juliana.campos@unesp.br (J.A.D.B.C.); bia.g.martins@unesp.br (B.G.M.); 2 School of Dentistry, São Paulo State University (UNESP), Campus Araraquara, São Paulo 14801-903, Brazil; lucas.campos@unesp.br; 3 William James Center for Research (WJCR), University Institute of Psychological, Social, and Life Sciences (ISPA), 1100-304 Lisbon, Portugal; jpmaroco@ispa.pt; 4 Mayo Clinic College of Medicine General Interne Medicine and International Medicine, Mayo Clinic, Rochester, MN 55905, USA; Saadiq.Rayya@mayo.edu; 5 Division of Maternal-Fetal Medicine, Department of Obstetrics and Gynecology, Mayo Clinic, Rochester, MN 55905, USA

**Keywords:** pandemic, COVID-19, mental health, quarantine

## Abstract

Background: Isolation measures used to contain epidemics generate social interaction restrictions and impose changes in routines of the public that increase negative psychological outcomes. Anxiety and depression are the most common symptoms. Objective: To evaluate the mental health of the Brazilian population during the SARs-CoV-2 pandemic and its relationship with demographic and health characteristics. Methods: Adults from all Brazilian States participated (*n* = 12,196; women: 69.8%, mean age = 35.2 years). The Depression, Anxiety and Stress Scale, and the Impact of Event Scale–revised were used (online survey). Data validity and reliability were verified by confirmatory factor analysis and ordinal alpha coefficient. The probability of presenting psychological symptoms was calculated by multiple logistic regression and odds ratio (OR) (0 = without symptoms, 1 = with mild, moderate, and severe levels of symptoms). Results: High prevalence of depression (61.3%), anxiety (44.2%), stress (50.8%), and psychological impact (54.9%) due to the isolation experienced from the pandemic was found. Younger individuals (OR = 1.58–3.58), those that felt unsafe (OR = 1.75–2.92), with a previous diagnosis of mental health (OR = 1.72–2.64) and/or had general health problems before the pandemic (OR = 1.17–1.51), who noticed changes in their mental state due to the pandemic context (OR = 2.53–9.07), and excessively exposed to the news (OR = 1.19–2.18) were at increased risk of developing symptoms. Women (OR = 1.35–1.65) and those with lower economic status (OR = 1.38–2.69) were more likely to develop psychological symptoms. Lower educational levels increased the likelihood of depressive (OR = 1.03–1.34) and intrusive symptoms (OR = 1.09–1.51). *Conclusions*: The pandemic and related factors can have a high impact on the mental health of the population. Demographic characteristics can influence the occurrence of psychological symptoms.

## 1. Introduction

Isolation and quarantine measures (used or experienced) during epidemics generate separation and restriction of human movement, imposing drastic changes in routine and the need for adaptation at a time of great physical, social, economic and psychological vulnerability. Despite all the efforts towards containing the spread of the disease, isolation and quarantine measures come with psychological costs to individuals and, therefore, some care and attention related to mental health must be provided [1,2,3,4,5,6,7]. The World Health Organization declared the new coronavirus disease (COVID-19) a pandemic on 11 March 2020. At the end of that month, due to the rapid increase of cases (or infections), Brazil declared a mandatory quarantine, excluding essential service workers.

The psychological consequences of quarantine and isolation measures have already been identified in previous epidemics such as SARS [8,9,10,11], Ebola [12,13], and H1N1 [14,15]. So far, most of the scientific information on the impact of the SARs-CoV-2 pandemic on mental health has been presented as letters to editors or brief reports from experts due to the ongoing nature of the pandemic and little data is available. The existing documents indicate that the current quarantine and mass social isolation can have concerning psychological effects [3,5,6,7,16]. As of July 2020, there were few published epidemiological studies focusing on the Chinese population, with some assessing the effects of the disease from a clinical point of view, including mental health in patients who contracted the disease [17,18] and the mental health effects on frontline healthcare workers highlighting their vulnerable situation [19,20]. Only four studies evaluated the mental health conditions on the general population—three from China and one from Brazil.

Gao et al. [21] investigated 4827 Chinese adults and reported a high prevalence of depression (48.3%), anxiety (22.6%), and concomitant depression and anxiety (19.4%). Wang et al. [22] reported that 16.5% of the 1210 Chinese respondents had symptoms of moderate to severe depression, 28.8% had severe anxiety, and 8.1% had moderate to severe stress levels. Figueiras et al. [23] carried out a study with 1460 Brazilian adults and reported that women, younger people, and those with less education had higher levels of depression and anxiety. The authors also describe behaviors of individuals during quarantine and their relationship to depression, anxiety, and stress symptoms. However, none of these studies evaluated a nationwide population. To date, only one nation-wide study has been carried out in China by Qiu et al. [24] with 52,730 participants from 36 provinces that found that peri-traumatic stress was related to sex, age, educational level, region of residence, local health structure, and being a migrant worker. Nevertheless, some areas of that large country were not included in the study.

The literature on COVID-19 is increasing in an exponential rate. Scientists around the world have been making efforts to understand the pandemic from different aspects. However, everyone has the same challenge, which is to provide quality responses as quickly as possible to the population. Science is being carried out in real time. In early September 2020, four more studies involving a general population sample (one Italian study [25], one Israeli [26], one from the United States [27] and one from United Kingdom [28]) were published with mental health data in the pandemic. Moccia et al. [25] conducted a study with 500 adults in the initial phase of the outbreak of SARs-CoV-2 in Italy (April). Of the participants, 62.0% reported not having psychological distress due to the pandemic, 19.4% had mild distress and 18.6% had moderate or severe, with women being the most affected. Individuals with cyclothymic, depressive and anxious temperaments were more likely to have moderate or severe psychological distress. Palgi et al. [26] conducted a study with 1059 adults to evaluate symptoms of depression and anxiety related to the SARs-CoV-2 pandemic in Israel. Most of the participants were women and had a high level of education. The youngest were more vulnerable to depressive and anxious symptoms and there was no relationship between these symptoms and the existence of pre-existing chronic diseases. Loneliness caused by isolation measures was the most prominent risk factor for the development of depressive and anxious symptoms.

Bruin [27] investigated the relationship between age and perceptions of risk, anxiety and depression during the pandemic in the United States. Data collection was carried out in March and 6,666 North American adults participated (52% women with an age range from 18 to 100 years [mean = 48.6; standard deviation = 16.6]). Older individuals had a greater perception of the risk of dying if they were infected with SARs-CoV-2, greater awareness that being in isolation reduces the risk of contagion and were less likely to have depression and anxiety.

To date, the UK study [28] appears to be the only longitudinal study that assessed changes in mental health in adults before and during the pandemic lockdown. The authors used data from the UK Household Longitudinal Study (UKHLS) which started in 2014, with surveys carried out at the beginning (for example, 1 January 2014) and at the end of two years (for example, 31 December 2015) with annual overlap. 15,376 individuals over the age of 16 from Wales, Scotland, Northern Ireland, and region of England participated. The mental distress increased from 18.9% (in 2018–2019) to 27.3% one month after the lockdown (April 2020). The current mean score for non-specific mental distress in the population was above expectations considering the estimated trend from 2014 to 2018. This increase was significantly higher among younger people, women, those with lower economic status and people living with children.

In a few days, new studies have been published with regards to the mental health impact during the COVID-19 pandemic. Among the recent studies, there is a systematic review/meta-analysis published by Salari et al. [29] that included 17 studies from 10 different countries. Among the most common psychological symptoms related to epidemics are post-traumatic stress [14], anxiety, and depression [1,4,6,22], which can be found during the isolation/quarantine period and can outlast the epidemic. Brooks et al. [1] highlight that this is due to the presence of specific stressors including the duration of the quarantine, fear of being infected, frustration and anger for the loss of the normal routine and reduction of physical and social contact, inadequate supply of food, water or accommodation, and confusing or inadequate information coming mainly from government and public agencies. Among the post-pandemic stressors, financial difficulties and social stigma towards infected individuals and health care workers are mentioned.

With a pandemic scenario, all individuals, to a greater or lesser degree, will depend on their mental resources to cope with the lifestyle changes, fears, and uncertainties. As the SARS-Cov2 pandemic, combined with the political and economic impact, imposes a new and distressing context, the monitoring of the population’s mental health may contribute to establish individual or collective strategies of support, guidance, prevention, and intervention to minimize mental trauma during and after the pandemic. Still, Sani et al. [16] emphasize that the identification of possible predictors for the psychological impact of the pandemic on populations can be relevant for the elaboration of more targeted and resolute intervention plans.

This nationwide study was carried out to assess aspects related to the mental health of the Brazilian population during the coronavirus pandemic. The prevalence of depression, anxiety and stress symptoms and their relationship with demographic and context-related characteristics was verified.

We hypothesized that the prevalence of psychological symptoms is high in Brazil in view of sanitary, economic and political insecurity. We also hypothesized that younger people, women, people with previously diagnosed mental disorders and those most exposed to pandemic news are more likely to develop symptoms of depression, anxiety and stress and be psychologically impacted.

## 2. Methods

### 2.1. Study Design and Sample

This was a cross-sectional observational study. Data were collected online using Google Forms with the links to the form sent by email, WhatsApp, or social networks. Brazilian individuals over 18 years of age were able to participate. The minimum sample size was estimated using α = 5%, *p* = 5% (prevalence of mortality from COVID-19), ε = 10%, and a loss rate of 25%. Thus, the minimum sample size was estimated at 9734. The sample was stratified by states to ensure a representation of all Brazilian States and the Federal District.

Information was collected on sex, age, state, monthly family income (1: below 240.00 USD; 2: between 240.00 USD and 383.00 USD; 3: between 384.00 USD and 1,652.00 USD; 4: between 1653.00 USD and 2153.00 USD; 5: above 2154.00 USD; 1 USD = 5.23 BRL; Available from: www.bcb.gov.br; Accessed 21 July 2020), number of people residing with the participant, time (minutes) spent per day watching or reading the news related to the pandemic, and education level (1: complete elementary I school, 2: complete elementary II school, 3: complete high school, 4: complete higher education and 5: complete graduate school). Further information was obtained on the mental health and general health problems diagnosed before the pandemic, mental health status during the pandemic, knowing someone who tested positive for COVID-19, considering COVID-19 as dangerous or not, sense of safe, and the opinion if the news about this viral pandemic is confusion. Information regarding the absence or presence of mental health problems was obtained by asking the participant if he had already received a medical diagnosis related to mental health disorders at some point before the pandemic. Regarding general health, the information was obtained in the same way, but, asking about medical diagnosis related to health in general. In addition, the participants were asked if they noticed any changes in their mental health status after the pandemic began, felt safe or unsafe in view of the pandemic scenario, believed the coronavirus was dangerous or not and if they considered the news transmitted about the pandemic confusing or adequate/clarifying.

Respondents also completed the Depression, Anxiety and Stress Scale (DASS-21) [30,31] and the Impact of Event Scale-revised (IES-R) [32]. The time frame for psychological impact was considered the period since the beginning of the pandemic in Brazil (11 March 2020).

### 2.2. Procedures and Ethical Aspects

For data collection, a non-probabilistic snowball sampling method was used. The first contacts took place on 18 May 2020, with professors from different higher education institutions in Brazil using e-mails available on the institutions’ websites. Invitations were also sent the official email addresses of the Slums Association (Central Única das Favelas (CUFA)) and seven non-governmental organizations (NGOs) in the country. The selected institutions were among the ten largest in the country and involved individuals of varying socioeconomic levels. To expand the sample, invitees were asked to pass on the survey link to their personal contacts. The researchers provided guidelines for distributing the link via email, WhatsApp, or social media. Data collection ended on 25 June 2020.

In Brazil, on 11 March 2020, social isolation measures began to be established. Six days later (March 17) the first death due to COVID-19 was confirmed, which contributed to the maintenance of the quarantine that lasted approximately until the beginning of July. After this period, there was a relaxation of social isolation, with a gradual reopening of trade and a recovery of the economy. During the 39-day period of data collection, the Brazilian Ministry of Health reported 973,894 cases of Covid-19 with 38,179 deaths due to the disease. At the beginning of the collection (May 18th) there were 254,220 cases and 16,792 deaths and on the last day of the collection (June 25th) there were 1,228,114 cases and 54,971 deaths due to COVID-19 reported in Brazil. During the data collection period, the highest number of COVID-19 deaths in 24 h in the country (*n* = 1.473) was reached on 4 June and the highest number of new cases in 24 h (*n* = 54.771) was observed on the 19th of June.

All subjects gave their informed consent for inclusion before they participated in the study. The study was conducted in accordance with the Declaration of Helsinki, and the protocol was approved by the National Research Ethics Commission of the Ministry of Health (CONEP) (C.A.A.E. 30604220.4.0000.0008).

### 2.3. Measuring Instruments

Symptoms of depression, anxiety, and stress were assessed using the Portuguese version of the DASS-21 [30,31]. The scale consists of 21 items distributed in three factors with a 4-point Likert-type response scale from 0 to 3 (0: did not apply to me at all, 1: some of the time, 2: a good part of time, 3: most of the time). The scores were calculated by adding the items’ response of each subscale and the participants were classified using the cutoff points proposed by Lovibond & Lovibond [33], multiplying the sum of the responses by two (Depression: Normal—0 to 9, Mild—10 to 13, Moderate—14 to 20, Severe—21 to 27, and Extremely severe ≥ 28; Anxiety: Normal—0 to 7, Mild—8 to 9, Moderate—10 to 14, Severe—15 to 19, and Extremely severe ≥ 20; Stress: Normal—0 to 14, Mild—15 to 18, Moderate—19 to 25, Severe—26 to 33, and Extremely severe ≥ 34).

The degree of psychological impact related to the pandemic was identified using the Portuguese version of the IES-R [32]. The IES-R is composed of 22 items distributed in three factors (avoidance, intrusion, and hyperarousal) with a Likert-type response scale of 5 points (0—not at all, 1—slightly, 2—moderately, 3—very and 4—extremely). IES-R calculated by the sum of responses to all items on the scale) and the recommendation by Wang et al. [22] (Normal—0 to 23; Mild—24 to 32; Moderate—33 to 36; Severe—≥37) were proposed to estimate the prevalence of psychological impact and its degree of involvement. Next, the cutoff points were transposed, considering the corresponding percentiles, for each factor of the scale so that one can also estimate the prevalence and the degree of involvement for the three factors of the IES-R separately (Avoidance and Intrusion: Normal—0 to 8; Mild—9 to 11; Moderate—12 to 13; Severe—≥14; Hyperstimulation: Normal—0 to 6; Mild—7 to 8; Moderate—9 to 10; Severe—≥11).

### 2.4. Data Validity and Reliability Indicators

Confirmatory factor analysis (AFC) was performed with the weighted least squares means and variance adjusted (WLSMV). The fit of the theoretical models to the data was assessed using the Comparative Fit Index (CFI), Tucker-Lewis Index (TLI), and Root Mean Square Error of Approximation (RMSEA) with a 90% confidence interval. The fit was considered adequate if CFI and TLI ≥ 0.90, and RMSEA ≤ 0.10 [34]. The factor loadings (λ) of the items were considered adequate if λ ≥ 0.50. Reliability was analyzed by the alpha ordinal coefficient (α) (adequate reliability: α ≥ 0.70). The analyses were conducted for the total sample (Brazil) and for each region of the country in order to identify possible model fit problems due to cultural differences. The MPLUS 7.2 program (Muthén and Muthén, Los Angeles, CA, USA) was used. The data were found to be valid and reliable and parameters are shown in Table 1. 

### 2.5. Statistical Analysis

Descriptive statistics were performed according to states. Then, an ordinal hierarchical model was developed to verify if the states’ variable affected the results. Two levels were considered, the individual and state subgroup. At the individual level, the level of impact by depression, anxiety, and stress and the psychological impact (avoidance, intrusion and hyperarousal) were considered as dependent variables. Sex, age, education, monthly income, previous mental and general health problems, sense of safety, number of people living with the participant, time of exposure to the news, frequency of socialization, knowing someone who tested positive for COVID-19, and change in mental health status after the start of the pandemic were independent variables. To verify the cluster effect, the intraclass correlation coefficient (ICC) was used. 

The prevalence of psychological symptoms were calculated according to sex (reference category (rc) = male), age group (rc: ≥ 55 years), number of people living with the participant, economic level (rc: < 240.00 USD), education level (rc: complete graduate school), sense of security in relation to the pandemic (rc: unsafe), previous health problems (rc: no), frequency of socialization (rc: equal to or greater than usual), prior mental illness (rc: absent), change in mental state due to the pandemic (rc: no), knowing someone who tested positive for COVID-19 (rc: no), time spent with the news (rc: < 60 min). A multiple logistic regression model was constructed and odds ratio (OR) per point and 95% confidence interval were calculated. The dependent variables (psychological symptoms) were grouped into absent (normal category = 0) and present (symptom present in some level of impact); time spent with the news was categorized according to 25th, 50th, and 75th percentiles (1: < 60 min; 2: 60–90 min; 3: 90–150 min; 4: ≥ 150 min) and age by the 25th, 50th, 75th and 90th percentiles (1: < 24; 2: 24–33; 3: 33–43; 4: 43–55; 5: ≥ 55 years). The significance level was 5%. The analyses were performed using the IBM SPSS Statistics v.22 software (IBM Corp, Armonk, NY, USA).

## 3. Results

A total of 13,584 people completed the questionnaires, but 95 were excluded for not meeting the inclusion criteria (61 were < 18 years of age and 34 were non-Brazilians). 1388 participants did not complete all items of the scales (DASS-21 and IER-S) and were excluded. Thus, the loss rate (considering individuals who did not respond to all the questions) was 10.2%, being below the expected from the sample size of the initial study (25%).

Of the included participants, 8.7% reported having an average monthly family income below 240.00 USD, 11.6% between 240.00 USD and 383.00 USD, 38.0% between 384.00 USD and 1652.00 USD, 16.1% between 1653.00 USD and 2153.00 USD, and 25.0% above 2154.00 USD. Regarding education, 50.1% had completed graduate studies, 20.7% had completed higher education, 28.8% had completed high school, and 0.4% had completed middle school. All Brazilian states were adequately represented (Table 2): 38.3% (*n* = 4677) were from the Southeast, 31.2% (*n* = 3804) from the Northeast, 12.3% (*n* = 1498) from the South, 9.8% (*n* = 1191) from the North, and 8.4% (*n* = 1026) from the Midwest.

There was a greater prevalence of women (69.8%) and younger people (50% < 33 years old and 10% ≥ 55 years) in the sample. The prevalence of previous mental health ranged from 20 to 43.1%. 81.5 to 95.2% of participants reported the appearance of some mental health symptom and 58.1 to 75.0% reported changes in their mental health status after the onset of the pandemic. Among individuals who had no previous medical diagnosis related to mental disorders (*n* = 8178), 85.5% reported the appearance of symptoms of psychological impairment after the start of the pandemic. Among individuals with a medical diagnosis of mental disorder, 96.2% reported new symptoms after the start of the pandemic. Considering the total sample, approximately 88.8% had some new symptoms after the start of the pandemic. A large number of people reported knowing someone who had tested positive for COVID-19 (68.8%) and most believed the coronavirus is a serious condition (97.9%) and felt unsafe about the current pandemic scenario (84.4%). Approximately one-third of participants found the broadcasted news confusing and the average time spent per day with news related to the pandemic was 125.2 (standard deviation = 128.9) min.

A cluster effect by states was not observed in the data, i.e., data variance due to the state of residence was very small (ICC for Depression = 0.050, Anxiety = 0.055, Stress = 0.054, Psychological impact related to the event = 0.025, Avoidance = 0.013, Intrusion = 0.017, Hyperarousal = 0.025). Thus, the subsequent analyzes will be presented for the whole of Brazil (*n* = 12,196).

Table 3 shows the prevalence of depressive symptoms, anxiety, stress and the psychological impact due to the pandemic according to the level of symptoms.

A high prevalence of depression, anxiety, stress, and psychological impact due to the pandemic was found in the population, especially at moderate and severe levels.

The occurrence of psychological symptoms according to demographic and pandemic-related characteristics is shown in Table 4. Younger people, those feeling unsafe in regards to the pandemic, with a previous mental health problem, who reported a change in their mental state, and who reported following the news to the greatest extent excessively follow the news had a higher risk of developing psychological symptoms. Women were more likely to develop psychological symptoms than men, except for depressive symptoms. The lower the economic level, the higher the chance of symptoms, except for stress. Lower education levels increased the likelihood of depressive and intrusive symptoms. The presence of previous health problems increased the chance of developing symptoms, with the exception of avoidance.

## 4. Discussion

This survey evaluated the mental health of Brazilians during the SARS-Cov-2 pandemic and identified characteristics that increased the risk of psychological symptoms. A high prevalence of depression (61.3%), anxiety (44.2%), stress (50.8%), avoidance (59.2%), intrusion (46.8%) and hyperarousal (50.1%) symptoms was observed. 

The increase in mental health symptoms in populations during epidemics is not a novelty [8,9,10,11,12,13,14,15], however, the current context deserves attention. In addition to being the largest pandemic in the last 100 years, the eradication of the disease is still a scientific and social challenge, and the effectiveness of control measures (such as hand hygiene, isolation, and quarantine) is thus still unknown,. Thus, the quarantine has been extended, with an unknown end date and the return to normal activities. In addition to the pandemic, the country’s economic, political, and social crises related to it contribute to a lack of feeling safe [3,4,5,6], creating an environment of multi-faceted vulnerability and unpredictability that affects the general population, especially in those who lack coping skills to manage the conflicts of this scenario.

The lack of feeling safe affected the majority of the study population (84.4%) and significantly increased the risk of developing psychological symptoms. This may be related to both the pandemic itself and the large volume of inaccurate and often conflicting information from the media and the government regarding the coronavirus disease and its control and treatment measures [1,5]. Gao et al. [21] concluded that the amount of false or manipulated information can generate unfounded fear and confuse people. The authors also reported that greater exposure to the news increases the chance of developing symptoms of depression and anxiety, which was also found in the present study. The time spent with the news was a protective factor for the avoidance symptom, indicating that individuals that are more exposed to the news (low avoidance behavior) could be aiming at acquiring the maximum information on the pandemic to gain some control of the situation. However, a false sense of control could aggravate the other psychological symptoms assessed in this study.

Less than one third of individuals reported medical diagnosis related to mental disorders before the pandemic. The prevalence of psychological symptoms among these individuals was extremely high (96.2%) after the beginning of the pandemic. In the total sample, 88.8% of people reported the appearance of one or more psychological symptoms after the beginning of the pandemic. It should be noted that this information was self-reported by the participants. These results are especially important, as they may suggest difficulties in dealing with emotional reactions in the context of the pandemic, whether due to the lack of coping strategies, the unpredictability of the new condition, and/or the feeling of vulnerability. Cullen et al. [35] suggest that psychological intervention measures be provided for the COVID-19-affected communities in primary and emergency healthcare services, especially for people with previous psychological problems. 

Actions to raise awareness on mental disorders and help symptom diagnosis are also suggested to reduce psychological distress and prevent new mental health problems [24,35]. In addition, Holmes et al. [2] highlight that individuals with previous symptoms are more vulnerable and have increased risk of physical and psychological effects from the pandemic [35], which was also observed in this study. Moccia et al. [25] point out that people with a depressive or anxious temperament are also more vulnerable. A striking finding in our study was that 85% of those who reported never having received a medical diagnosis related to psychological problems, reported appearance of symptoms during the pandemic, corroborating the findings by Cullen et al. [35] Furthermore, the presence of moderate to severe symptoms of psychological impact (IES-R) can indicate a risk for future development of post-traumatic stress disorder. Therefore, mental health care must be expanded to restrain these symptoms and prevent their aggravation.

In accordance to the literature, psychological symptoms were more common among younger individuals and women [22,23,24,25,26,27,28,29]. The uncertainty of the future, school and university closings and new teaching formats, the breakdown of interpersonal relationships and an immature cognitive and behavioral repertoire to cope with the pandemic demands could explain the greater risk of psychological symptoms in young adults. The results presented by Palgi et al. [26] point out that the feeling of loneliness due to social isolation is greater among young people, which can increase the risk of developing depressive and anxious symptoms. Still, Bruin [27] showed that older individuals report less negative emotions, have better mental health and less responsiveness to daily stressors, which favors a lesser experience of symptoms of depression and anxiety. To explain why stressful factors have a greater impact on women, Almeida and Kessler [36] suggest that women tend to ruminate negative thoughts more, prolonging distress, increasing the effects of stressors on mood, interfering with behaviors, and impeding the development of a strategies to eliminate stressors. Moccia et al. [25] suggest that women’s greatest vulnerability to psychological distress is related to genetic, socio-cultural, hormonal, and developmental factors.

The effect of the economic level on psychological symptoms was expected as people in disadvantaged economic and social situations are affected at a much greater extent due to lower access to health care, inability to quarantine without risking losing a job or decreasing income, among several other factors, making this group even more vulnerable.

This study was conducted on a large sample of individuals that covered the entire country and presented valid and reliable findings. However, some limitations should be reported. First, data collection was carried out online, which certainly resulted in more younger individuals and with higher economic level and education being selected. It should be remembered that older people and those with less economic power or education have lower access to or literacy in digital resources. However, this was the feasible strategy for gathering information during the pandemic. Sample selection could also have been biased by the snowball process starting within universities. Despite contacts with NGOs from populations with low income and education levels, the adherence of this group was not representative of the country population, which was confirmed by the absence of a cluster by States in a country with great inequalities among regions. Finally, the cross-sectional study design does not allow causality inference, and therefore, future longitudinal studies should the developed.

Another aspect to be reported is the absence of a national mental health survey that could be used to compare pre, peri and post-pandemic data and/or to monitor the mental health of the Brazilian population. It is expected that the impact generated by the current context of the pandemic may trigger the need to develop this type of tracking in a continuous and rigorous manner, as in the United Kingdom [28].

Despite these limitations, this is the first Brazilian nation-wide study to present quality evidence that deserves a careful look from government officials, managers, and mental healthcare professionals. The development of a national plan is suggested for epidemiological surveillance and minimization of the psychological impacts of the COVID pandemic in the population. As Qiu et al. [24] proposes, a nation-wide program for coordinating psychological first aid [potentially using telemedicine] and establishing a prevention and intervention system could be highly effective. The psychological and psychiatric needs cannot be neglected during any phase of pandemic management, as they play an important role in public policies adherence and dealing with infection threats and possible losses [34]. Moreover, attention to mental care provided during the crisis can help in the reconstruction process of all individuals and provide the skills needed for the post-pandemic world.

## 5. Conclusions

A high prevalence of mental health symptoms was found in the Brazilian adult population during the SARS-Cov2 pandemic. The impact level of symptoms of depression, anxiety, and stress, and the impact of factors directly related to the current context, emphasizes the importance of mental health care. The probability of having psychological symptoms was affected by several sample characteristics (e.g., sex, age, previous diagnosis of mental health-related disorder, individuals most exposed to pandemic news), which must be considered in the development of support, prevention, and treatment strategies.

## Figures and Tables

**Table 1:** Psychometric parameters for fit of the models to the samples of the instruments used (Depression, Anxiety and Stress Scale (DASS-21), and Impact of Event Scale-revised, (IES-R)).

Instrument	Sample	*n*	Confirmatory Factor Analysis *				
			λ	CFI	TLI	RMSEA (90% CI)	α
DASS-21	Brazil	12,196	0.54–0.92	0.978	0.975	0.065 (0.064–0.066)	0.892–0.947
	Midwest	1026	0.52–0.92	0.973	0.970	0.068 (0.064–0.072)	0.879–0.944
	Northeast	3804	0.53–0.91	0.977	0.974	0.066 (0.064–0.067)	0.890–0.947
	North	1191	0.52–0.91	0.974	0.971	0.070 (0.066–0.074)	0.887–0.945
	Southeast	4677	0.55–0.93	0.980	0.977	0.064 (0.062–0.066)	0.896–0.949
	South	1498	0.55–0.93	0.981	0.978	0.059 (0.056–0.063)	0.892–0.941
IES-R ^†^	Brazil	12,196	0.51–0.89	0.964	0.960	0.072 (0.071–0.073)	0.879–0.927
	Midwest	1026	0.53–0.89	0.968	0.964	0.067 (0.063–0.071)	0.883–0.928
	Northeast	3804	0.52–0.89	0.965	0.961	0.072 (0.070–0.074)	0.878–0.928
	North	1191	0.53–0.88	0.965	0.960	0.069 (0.065–0.072)	0.883–0.926
	Southeast	4677	0.50–0.89	0.964	0.959	0.073 (0.071–0.075)	0.877–0.926
	South	1498	0.50–0.89	0.963	0.958	0.072 (0.069–0.075)	0.871–0.924

* Comparative Fit Index (CFI), Tucker-Lewis Index (TLI), and Root Mean Square Error of Approximation (RMSEA) with a 90% confidence interval; ^†^ With fit refinement.

**Table 2:** Population (*N*) according to the State and minimum sample size estimated (*n*), final sample size (*n*’), and demographic characteristics.

Abbreviation, State, Region	*N* *	*n*	*n*’	Mean (SD; min/max)		%							
				Age in Years	Number of People in the Residence	Women	COVID-19 Seriousness	Previous Mental Problem	Mental Symptom during Pandemic	Change in Mental Health during Pandemic	Know COVID-19 Positive People	Feeling Unsafe	News is Confusing
AC—Acre (North)	881,935	41	46	37.7 (8.3; 18/60)	3.0 (1.2; 1–6)	50.0	97.8	20.0	93.5	67.4	95.7	91.3	32.6
AL—Alagoas (Northeast)	3,337,357	155	199	35.6 (12.2; 18/70)	3.3 (1.5; 1/13)	60.6	100.0	24.0	88.4	66.8	91.0	89.4	30.7
AM—Amazonas (North)	4,144,597	192	201	38.2 (13.1; 18/69)	3.4 (1.7; 1/15)	62.5	97.5	25.6	87.6	65.7	96.5	81.6	34.8
AP—Amapá (North)	845,731	39	69	37.6 (11.5; 18/65)	3.3 (1.7; 1/10)	68.1	100.0	26.9	91.3	73.9	98.6	89.9	29.0
BA—Bahia (Northeast)	14,873,064	689	723	36.8 (12.3; 18/80)	3.0 (1.5; 1/15)	70.7	99.0	31.6	90.7	66.5	73.6	86.3	34.6
CE—Ceará (Northeast)	9,132,078	423	430	34.7 (12.3; 18/70)	3.5 (1.5; 1/10)	68.6	97.0	25.0	91.1	72.6	89.1	85.3	29.3
DF—Distrito Federal (Midwest)	3,015,268	140	256	35.3 (13.1; 18/73)	3.22 (1.3; 1/7)	72.7	98.4	43.1	89.1	75.0	68.8	86.3	39.5
ES—Espírito Santo (Southeast)	4,018,650	186	199	30.0 (10.5; 18/70)	3.3 (1.4; 1/9)	80.4	98.0	32.7	88.9	71.9	68.8	83.4	36.2
GO—Goiás (Midwest)	7,018,354	325	374	33.8 (11.9; 18/66)	3.3 (1.4; 1/10)	77.3	96.5	35.3	89.0	67.9	57.8	84.0	46.5
MA—Maranhão (Northeast)	7,075,181	328	1196	29.5 (10.4; 18/67)	3.8 (1.7;1/10)	65.4	98.2	24.3	89.7	68.5	94.1	84.1	36.4
MG—Minas Gerais (Southeast)	21,168,791	980	1006	33.8 (12.5; 18/72)	3.2 (1.3; 1/9)	71.3	98.8	35.2	89.8	67.2	44.5	84.7	37.1
MS—Mato Grosso do Sul (Midwest)	2,778,986	129	230	35.4 (11.2; 18/66)	3.0 (1.3; 1/7)	57.8	96.5	30.5	86.0	64.3	40.4	84.3	33.2
MT—Mato Grosso (Midwest)	3,484,466	161	166	33.0 (11.1; 18/62)	3.2 (1.5; 1/10)	63.9	92.2	31.3	84.3	58.4	46.4	81.3	36.6
PA—Pará (North)	8,602,865	398	442	35.3 (12.4; 18/94)	3.5 (1.6; 1/11)	67.6	98.2	23.7	93.4	71.7	97.7	86.0	37.8
PB—Paraíba (Northeast)	4,018,127	186	186	38.3 (11.7; 18/70)	3.2 (1.6; 1/11)	65.6	98.4	24.9	89.8	67.7	86.6	81.2	34.9
PE—Pernambuco (Northeast)	9,557,071	443	429	37.0 (12.1; 18/78)	3.3 (1.6;1/15)	63.9	98.6	24.6	90.1	64.3	88.6	85.8	34.3
PI—Piauí (Nordeste)	3,273,227	152	222	35.7 (10.6; 18/66)	3.6 (1.5; 1/8)	68.0	99.5	30.6	89.6	68.9	82.9	88.3	29.9
PR—Paraná (South)	11,433,957	530	536	39.3 (12.9; 18/72)	3.0 (1.3; 1/10)	71.5	95.9	36.7	88.8	64.4	50.6	81.5	35.0
RJ—Rio de Janeiro (Southeast)	17,264,943	800	868	38.3 (13.5; 18/78)	3.0 (1.4; 1/13)	69.7	98.2	30.2	88.4	62.7	88.5	85.3	34.2
RN—Rio Grande do Norte (Northeast)	3,506,853	162	167	29.0 (9.5; 18/64)	3.8 (1.6; 1/9)	66.5	98.2	29.9	95.2	70.1	74.9	80.8	31.1
RO—Rondônia (Norte)	1,777,225	82	124	37.1 (11.7; 19/69)	3.4 (1.8;1/10)	71.0	98.4	24.6	81.5	58.1	82.3	88.7	38.7
RR—Roraima (North)	605,761	28	239	28.3 (10.7; 18/68)	3.9 (1.9; 1/15)	67.8	97.9	30.6	89.5	74.9	81.2	87.0	36.8
RS—Rio Grande do Sul (South)	11,377,239	527	582	38.5 (14.0; 18/72)	2.8 (1.2; 1/8)	75.6	97.8	35.2	87.6	65.8	41.4	80.8	30.2
SC—Santa Catarina (South)	7,164,788	332	380	35.0 (12.8; 18/68)	2.9 (1.3; 1/10)	65.8	97.6	33.2	86.5	68.9	47.6	84.7	39.3
SE—Sergipe (Northeast)	2,298,696	106	252	31.2 (10.4; 18/64)	3.5 (1.6; 1/11)	70.2	99.6	26.7	90.4	68.3	77.4	88.8	37.7
SP—São Paulo (Southeast)	45,919,049	2127	2604	36.5 (14.8; 18/82)	3.1 (1.4; 1/10)	73.4	97.7	34.5	87.6	64.6	55.1	83.5	40.4
TO—Tocantins (North)	1,572,866	73	70	38.2 (10.3; 20/64)	3.0 (1.5; 1/8)	67.1	95.7	38.6	84.3	71.4	61.4	81.4	41.4
Total	210,147,125	9734	12,196	35.2 (13.0;18/94)	3.2 (1.50; 1/15)	69.8	97.9	31.2	88.9	66.8	68.8	84.4	36.5

* Brazilian Geography and Statistics Institute—IBGE: State population.

**Table 3:** Prevalence (%) of symptoms of depression, anxiety and stress and the psychological impact due to the pandemic according to level of symptoms in Brazilian adults (*n* = 12,196).

		Level (%)			
Instrument	Factor	Normal	Mild	Moderate	Severe or Extremely Severe
DASS-21	Depression	38.7	14.5	21.8	25.0
	Anxiety	55.8	8.5	19.2	16.5
	Stress	49.2	15.5	16.9	18.4
IES-R	Psychological impact	45.1	19.7	7.2	28.0
	Avoidance	40.2	18.0	10.9	30.9
	Intrusion	53.2	14.3	7.1	25.3
	Hyperarousal	49.9	12.4	10.0	27.7

**Table 4:** Odds ratio (OR) with 95% confidence interval (95% CI) for psychological symptoms (0 = Absent/1 = Present) according to characteristics of interest.

	Symptom OR (IC 95%)						
Characteristic	Depression	Anxiety	Stress	Psychological Impact-General	Avoidance	Intrusion	Hyperarousal
Sex Male/Female *	1.10 (0.99–1.22)	1.40 ^†^ (1.26–1.54)	1.651 (1.49–1.83)	1.50 ^†^ (1.36–1.66)	1.57 ^†^ (1.43–1.72)	1.35 ^†^ (1.22–1.49)	1.40 ^†^ (1.26–1.55)
Age (≥55 years *) < 24	2.98 ^†^ (2.38–3.74)	1.78 ^†^ (1.42–2.23)	3.58 ^†^ (2.82–4.53)	2.52 ^†^ (2.02–3.14)	1.75 ^†^ (1.43–2.14)	1.58 ^†^ (1.27–1.97)	3.03 ^†^ (2.40–3.81)
24–33	2.05 ^†^ (1.72–2.45)	1.47 ^†^ (1.21–1.78)	2.56 ^†^ (2.10–3.12)	1.76 ^†^ (1.47–2.10)	1.27 ^†^ (1.08–1.49)	1.41 ^†^ (1.18–1.70)	2.18 ^†^ (1.80–2.64)
33–43	1.49 ^†^ (1.26–1.76)	1.21 (1.00–1.46)	1.93 ^†^ (1.59–2.34)	1.27 ^†^ (1.07–1.51)	1.00 (0.86–1.17)	1.06 (0.89–1.27)	1.54 ^†^ (1.28–1.85)
43–55	1.13 (0.95–1.35)	1.09 (0.90–1.33)	1.29 ^†^ (1.05–1.58)	1.12 (0.94–1.35)	0.92 (0.78–1.07)	0.99 (0.82–1.19)	1.24 (1.01–1.50)
N people in the residence	0.96 (0.92–0.99)	1.06 (1.03–1.10)	1.02 (0.98–1.05)	1.02 (0.99–1.05)	1.01 (0.98–1.04)	1.00 (0.97–1.03)	1.04 (1.01–1.08)
Economic level ^‡^ (>2154.00 USD *) < 240.00 USD	1.68 ^†^ (1.34–2.90)	2.69 ^†^ (2.20–3.28)	1.18 (0.96–1.46)	1.92 ^†^ (1.56–2.37)	1.38 ^†^ (1.14–1.67)	1.82 ^†^ (1.49–2.22)	1.91 ^†^ (1.55–2.36)
Between 240.00 USD and 383.00 USD	1.70 ^†^ (1.40–2.06)	2.47 ^†^ (2.07–2.95)	1.25 (1.03–1.50)	1.66 ^†^ (1.39–1.99)	1.55 ^†^ (1.31–1.84)	1.44 ^†^ (1.21–1.71)	1.60 ^†^ (1.33–1.92)
Between 384.00 USD and 1652.00 USD	1.37 ^†^ (1.20–1.55)	1.72 ^†^ (1.52–1.96)	1.08 (0.95–1.23)	1.42 ^†^ (1.26–1.61)	1.29 ^†^ (1.15–1.45)	1.26 ^†^ (1.12–1.43)	1.28 ^†^ (1.12–1.45)
Between 1653.00 USD and 2153.00 USD	1.13 (0.98–1.29)	1.31 ^†^ (1.13–1.51)	1.12 (0.97–1.30)	1.17 ^†^ (1.01–1.34)	1.22 ^†^ (1.07–1.38)	1.09 (0.94–1.25)	1.17 (1.01–1.36)
Education (completed graduate studies *) Up to Completed High School	1.55 ^†^ (1.30–1.84)	1.12 (0.96–1.32)	1.15 (0.97–1.36)	1.12 (0.95–1.32)	1.04 (0.89–1.21)	1.29 ^†^ (1.09–1.51)	1.13 (0.95–1.33)
Completed Higher Education	1.17 ^†^ (1.03–1.34)	1.02 (0.90–1.16)	0.99 (0.86–1.13)	1.02 (0.90–1.16)	1.01 (0.90–1.14)	1.05 (0.93–1.19)	1.00 (0.88–1.14)
Feel Unsafe/Safe *	2.52 ^†^ (2.22–2.87)	2.16 ^†^ (1.87–2.50)	2.70 ^†^ (2.33–3.13)	2.48 ^†^ (2.17–2.84)	1.75 ^†^ (1.56–1.97)	2.92 ^†^ (2.52–3.38)	2.67 ^†^ (2.31–3.09)
Health problems Yes/No *	1.17 ^†^ (1.05–1.30)	1.51 ^†^ (1.36–1.67)	1.31 ^†^ (1.18–1.47)	1.34 ^†^ (1.21–1.49)	1.08 (0.98–1.19)	1.42 ^†^ (1.28–1.57)	1.44 ^†^ (1.30–1.61)
Socialization Lower/equal or higher than usual *	1.14 (1.00–1.29)	1.08 (0.96–1.23)	0.99 (0.87–1.12)	1.03 (0.91–1.17)	0.99 (0.89–.11)	1.03 (0.91–1.17)	1.02 (0.90–1.16)
Previous mental disorder Yes/No *	2.38 ^†^ (2.14–2.65)	2.64 ^†^ (2.39–2.91)	2.58 ^†^ (2.32–2.87)	2.41 ^†^ (2.18–2.67)	1.72 ^†^ (1.56–1.89)	2.42 ^†^ (2.19–2.66)	2.57 ^†^ (2.32–2.85)
Mental health change due to pandemic Yes/No *	4.99 ^†^ (4.52–5.50)	5.19 ^†^ (4.65–5.80)	9.07 ^†^ (8.10–10.15)	5.06 ^†^ (4.58–5.59)	2.53 ^†^ (2.31–2.78)	5.09 ^†^ (4.58–5.66)	6.55 ^†^ (5.87–7.29)
Know people positive for COVID-19 (Yes/No *)	1.14 ^†^ (1.03–1.26)	0.95 (0.87–1.05)	0.97 (0.88–1.08)	0.86 (0.78–0.95)	0.93 (0.85–1.02)	0.90 (0.82–0.99)	0.88 (0.80–0.97)
Time spent with news (<60 min *) 60–90	1.11 (0.97–1.27)	0.97 (0.85–1.11)	1.01 (0.88–1.17)	0.88 (0.77–1.01)	0.80 ^†^ (0.71–0.91)	1.09 (0.96–1.25)	1.08 (0.94–1.24)
90–150	1.33 ^†^ (1.15–1.53)	1.09 (0.94–1.25)	1.05 (0.90–1.22)	1.04 (0.90–1.20)	0.74 ^†^ (0.65–0.84)	1.48 ^†^ (1.28–1.70)	1.42 ^†^ (1.23–1.64)
≥150	1.67 ^†^ (1.44–1.93)	1.41 ^†^ (1.23–1.63)	1.45 ^†^ (1.25–1.69)	1.19 ^†^ (1.04–1.37)	0.66 ^†^ (0.58–0.75)	2.18 ^†^ (1.90–2.51)	1.83 ^†^ (1.59–2.12)

* Reference category; ^†^ *p* < 0.001; ^‡^ 1 USD = 5.23 BRL.
